# The USP21/YY1/SNHG16 axis contributes to tumor proliferation, migration, and invasion of non-small-cell lung cancer

**DOI:** 10.1038/s12276-019-0356-6

**Published:** 2020-01-20

**Authors:** Pei Xu, Haibo Xiao, Qi Yang, Rui Hu, Lianyong Jiang, Rui Bi, Xueyan Jiang, Lei Wang, Ju Mei, Fangbao Ding, Jianbing Huang

**Affiliations:** Department of Cardiothoracic Surgery, Xin Hua Hospital, Shanghai Jiao Tong University School of Medicine, 200092 Shanghai, China

**Keywords:** Non-small-cell lung cancer, Ubiquitylation, Cell biology

## Abstract

Deubiquitinases (DUBs) and noncoding RNAs have been the subjects of recent extensive studies regarding their roles in lung cancer, but the mechanisms involved are largely unknown. In our study, we used The Cancer Genome Atlas data set and bioinformatics analyses and identified USP21, a DUB, as a potential contributor to oncogenesis in non-small-cell lung cancer (NSCLC). We further demonstrated that USP21 was highly expressed in NSCLCs. We then conducted a series of in vitro and in vivo assays to explore the effect of USP21 on NSCLC progression and the underlying mechanism involved. USP21 promoted NSCLC cell proliferation, migration, and invasion and in vivo tumor growth by stabilizing a well-known oncogene, Yin Yang-1 (YY1), via mediating its deubiquitination. Furthermore, YY1 transcriptionally regulates the expression of SNHG16. Moreover, StarBase bioinformatics analyses predicted that miR-4500 targets SNHG16 and USP21. A series of in vitro experiments indicated that SNHG16 increased the expression of USP21 through miR-4500. In summary, the USP21/YY1/SNHG16 axis plays a role in promoting the progression of NSCLC. Therefore, the USP21/YY1/SNHG16/miR-4500 axis may be a potential therapeutic target in NSCLC treatment.

## Lung cancer: Targeting a vicious circle

Therapies targeting a molecular feedback loop involved in tumor growth may prove valuable for treating non-small-cell lung cancer. Fangbao Ding, Jianbing Huang, and co-workers at Shanghai Jiao Tong University in Shanghai, China, have shown how an enzyme called USP21 promotes cancer cell proliferation and tumor growth in non-small-cell lung cancer. The team took cancerous and non-cancerous lung tissue samples from 42 patients, and analyzed the expression and behavior of USP21. The enzyme was highly expressed in cancerous tissues, where it stabilized a known gene with the potential to cause cancer called YY1. This gene also regulated the expression of a particular RNA molecule, which in turn worked to increase levels of USP21. This cyclical process encouraged the proliferation, migration and invasion of non-small-cell lung cancer cells, and may provide a future therapeutic target.

## Introduction

Lung cancer (LC) is a main cause of cancer-related death, with 85% of cases involving non-small-cell LC (NSCLC)^1–3^. Despite developments in targeted therapy and immunotherapy, the 5-year survival rate for advanced LC patients is still very low^4^. Such a low percentage of survival could be due to advanced local invasive capacity, metastasis, and recurrence^5^. Additionally, there is also a rising need for more rapid identification of the disease through the use of appropriate markers, and finding new markers requires identifying the mechanism of action during disease progression. The discovery of new molecular biological mechanisms is therefore of great significance for the diagnosis and treatment of NSCLC.

USP21 is a member of the deubiquitinase (DUB) family^6,7^, which catalyzes the hydrolysis of ubH2A^8^. A study showed that USP21 binds to the promoter of interleukin-8 (IL-8) to transcriptionally mediate its initiation^9^. Moreover, it has been reported that USP21 has a high expression level and exerts oncogenic effects in bladder and liver cancers^10,11^. USP21 deubiquitinates and stabilizes MEK2, which contributes to tumorigenesis^11^. In addition to MEK2, USP21 is known to deubiquitinate and stabilize other proteins, such as GATA3 and Gli1^12,13^. However, the role of USP21 in NSCLC is still unknown.

Yin Yang-1 (YY1) is a ubiquitously expressed zinc-finger transcription factor^14^. It plays a role in a series of biological functions, such as embryogenesis, cellular proliferation, differentiation, and tumorigenesis^14,15^. YY1 has been known to activate or inactivate the gene expression of other genes with roles in tumorigenesis, and it may be involved in the transcriptional regulation of 10% of the total mammalian gene set^16^. Moreover, it has been reported that YY1 has a high expression level in many types of cancer, such as gastric cancer and prostate cancer, and it promotes cancer cell proliferation and metastasis, thus acting as an oncogene in cancer development^17,18^. In addition, previous studies have shown that YY1 transcriptionally activated lncRNA-PVT1 by directly binding to its promoter region in LC^19^. However, the mechanism of YY1 in LC is still unclear and needs to be further elucidated.

Studies have shown that long noncoding RNAs (lncRNAs) are dysregulated in human cancers, play crucial roles in tumor development and progression, and are involved in various biological processes^20^. The hypothesis that lncRNAs might function as competing endogenous RNAs (ceRNAs, or as molecular sponges) to modulate microRNAs (miRNAs)^21^ has been verified by other studies. For example, lncRNA CASC7 inhibits the proliferation and migration of colon cancer cells via inhibiting miRNA-21^22^, and lncRNA FTX inhibits hepatocellular carcinoma metastasis and invasion by competitively recruiting miR-374a^23^. Specifically, lncRNA SNHG16 has been studied extensively in many different types of cancers, such as bladder cancer^24^, breast cancer^25^, cervical cancer^26^, gastric cancer^27^, and glioma^28^. Recently, a study showed that SNHG16 is highly expressed in NSCLC patients^29^. This study elucidated the role of SNHG16 in targeting miR-146 to thereby regulate the expression of MUC5AC. miRNAs are important targets of lncRNAs, and their expression is associated with a better prognosis in many different cancers. Specifically, one such miRNA of interest was miR-4500, which, when overexpressed, has been associated with a higher patient survival rate in NSCLC^30^. However, the function of these networks and their mechanisms upstream and downstream in association with the pathogenesis of NSCLC remain poorly understood.

Our current study, for the first time, reported that USP21 acted as an oncogene by deubiquitinating YY1 to stabilize its protein levels in NSCLC cells. We found that YY1 bound to the SNHG16 promoter region and transcriptionally upregulated the expression of SNHG16, which increased the expression level of USP21 through miR-4500. Therefore, the USP21/YY1/SNHG16 axis promoted NSCLC cell proliferation and invasion.

## Materials and methods

### Clinical samples

LC tissues and corresponding non-cancerous tissues (distance from the cancer area was >2 cm) were collected from 42 NSCLC patients at Xin Hua Hospital Affiliated to Shanghai Jiao Tong University School of Medicine (Shanghai, China). These patients were diagnosed with LC based on histopathological evaluations (shown in Table 1). None of these patients received any preoperative chemotherapy or radiation therapy. After surgical resection, all tissue samples were frozen in liquid nitrogen and stored at −80 °C until use. All procedures that involved human participants were conducted in accordance with the ethical standards of the institution and/or national research committee. All patients signed informed consent, and the study was approved by the Xin Hua Hospital.

**Table 1:** Correlation between USP21 expression and clinicopathological parameters of NSCLC patients (*n* = 42).

Clinicopathological parameters	*n*	Relative USP21 expression		
		Low	High	*p* Value
Age				0.612
≤60	26	16	10	
>60	16	7	9	
Sex				0.473
Male	31	14	17	
Female	11	6	5	
Smoker				0.078
Yes	25	11	14	
No	17	10	7	
Lymph node metastasis				0.035*
Positive	14	9	5	
Negative	28	11	17	
TNM stage				0.041*
I/II	26	14	12	
III/IV	16	5	11	
Tumor differentiation				0.246
Well/moderate	37	20	17	
Poor	5	2	3	
Histological tumor type				0.453
Adenocarcinoma	21	12	9	
Squamous cell carcinoma	14	6	8	
Others	7	4	3	

*χ*^2^ test. *p < 0.05

### Cell culture

The NSCLC cell lines (A549, H1299, NCI-H460, and NCI-H520) and the normal human lung epithelial cell line 16HBE were obtained from the Institute of Biochemistry and Cell Biology of the Chinese Academy of Sciences (Shanghai, China). The cells were cultured in RPMI-1640 medium supplemented with 10% fetal bovine serum (HyClone, Logan, UT, USA), 100 IU/mL penicillin, and 100 μg/mL streptomycin (HyClone), and they were maintained at 37 °C with 5% CO_2_. Cells at ~80% confluence were subcultured by using 0.25% trypsin solution (HyClone).

### Histology and immunostaining

Tumor tissues were fixed in formalin, embedded in paraffin, and sectioned into 5 µm slices for hematoxylin and eosin staining and immunohistochemistry (IHC). Tissue sections were rehydrated, and antigen retrieval was performed. Then, tissue sections were incubated with anti-USP21 antibody (1:100; Abcam, Cambridge, MA, USA) for 12 h at 4 °C. After multiple washes, the sections were incubated with goat anti-rabbit secondary antibody (1:500; Abcam) for 1 h at room temperature and then visualized using a 3,3′-diaminobenzidine solution (Sigma, St. Louis, MO, USA).

### Oligonucleotide and plasmid transfection

MiR-4500 mimics (50 nM), inhibitors (100 nM), and their negative controls were designed and synthesized by RiboBio (Guangzhou, China). Silencing experiments were performed using USP21 small interfering RNA (siRNA), YY1 siRNA, SNHG16 siRNA, and the control sequence; overexpression experiments were performed using pCMV-USP21, pCMV-YY1, pCMV-SNHG16, and the control vector. Experiments were performed in 96-well plates, and transfections were performed using Lipofectamine® 2000 (Invitrogen, Carlsbad, CA, USA). Forty-eight hours after transfection, the cells were harvested for experiments.

### RNA extraction and quantitative reverse transcription-PCR

Total RNA was extracted from tissues and cell lines using TRIzol reagent (Life Technologies, Carlsbad, CA, USA) according to the manufacturer’s instructions. One microgram of total RNA was used for reverse transcription. Quantitative reverse transcription-PCR (qRT-PCR) was performed using SYBR Green Master mix (Life Technologies). The relative gene expression was calculated using the 2^−ΔΔCt^ method. The primers used were as follows: USP21 forward, 5′-AGGTGTCTCTGCGGGATTGTT-3′ and reverse, 5′-CGATTCAGATGGAGCACGAGG-3′; YY1 forward, 5′-ACGGCTTCGAGGATCAGATTC-3′ and reverse, 5′-TGACCAGCGTTTGTTCAATGT-3′; SNHG16 forward, 5′-AGCTGCTCTGAACCAGGACCT-3′ and reverse 5′-CGCATGGCGATTACTTTAGAGG-3′; miR-4500 forward, 5′-CGGGTGAGGTAGTAGTTTCTTG-3′ and reverse 5′-GCAGGGTCCGAGGTATTC-3′; U6 forward, 5′-CTCGCTTCGGCAGCACA-3′ and reverse, 5′-AACGCTTCACGAATTTGCGT-3′; and GAPDH forward, 5′-ACTTTGGTATCGTGGAAGGACTCAT-3′ and reverse 5′-GTTTTTCTAGACGGCAGGTCAGG-3′.

### Western blot analysis

Total protein was isolated from NSCLC cells or tissues using enhanced RIPA lysis buffer (Thermo Scientific, Waltham, MA, USA). Equal amounts of total protein (10 μg) were separated by sodium dodecyl sulfate-polyacrylamide gel electrophoresis on a 12% gel and transferred to polyvinylidene difluoride membranes. Membranes were immersed in 5% nonfat milk for 1 h at room temperature to block nonspecific binding sites and then incubated overnight at 4 °C in the following primary antibodies obtained from Abcam: anti-USP21 (1:500), anti-YY1 (1:1000), anti-E-cadherin (1:500), and anti-N-cadherin (1:1000). Next, the membranes were incubated with horseradish peroxidase-conjugated secondary antibodies (1:1000, Abcam) for 2 h at room temperature. Protein bands were visualized by enhanced chemiluminescence, and expression was normalized to β-actin.

### MTT assay

Briefly, for MTT (3-(4,5-dimethylthiazol-2-yl)-2,5-diphenyltetrazolium bromide) assays, the cells were seeded at 2 × 10^3^ cells/well into 96-well plates. Transfected cells were cultured at 37 °C for 24, 48, 72, or 96 h and treated with 0.5 mg/mL MTT solution (Sigma-Aldrich, Shanghai, China) for 2 h. Purple crystals were dissolved by adding 100 μL of dimethyl sulfoxide. Cell viability was assessed by reading the absorbance at 570 nm with a microplate reader (Bio-Rad, Hercules, CA, USA).

### Migration and invasion assays

A total of 5 × 10^4^ cells prepared in serum-free medium were loaded into the upper compartment of an uncoated transwell chamber (Corning Life Sciences, Corning, NY, USA), which was used to assess migration, or a chamber coated with Matrigel (Corning) for invasion experiments, and complete medium was placed in the lower chamber as a chemo-attractant stimulus. After incubating for 24 h, cells on the upper surface of the filter were wiped out with a cotton swab. The cells migrating to the bottom surface of the filter were fixed in 4% paraformaldehyde, stained with 0.1% crystal violet, and counted under a microscope in three random fields per filter.

### Luciferase reporter assay

The SNHG16 promoter or the predicted miR-4500 binding sequence in SNHG16 and USP21 3′-untranslated region (UTR) were cloned into the pmirGlo reporter gene vector (Promega, Madison, WI, USA) and were verified by sequencing. The putative binding sites were mutated using a KOD-Plus Mutagenesis Kit (Toyobo Biochemicals, Osaka, Japan) to abolish their binding. Cells were transfected with Lipofectamine 2000 (Invitrogen) in 6-well plates, and luciferase activity was measured 48 h after transfection using a Dual Luciferase Reporter Assay Kit (Promega) according to the manufacturer’s protocol.

### Co-immunoprecipitation assay

The cells were harvested and treated with RIPA lysis buffer supplemented with 10 mM *N*-ethylmaleimide and a protease inhibitor and phosphatase inhibitor cocktail (Thermo Scientific). Next, the cells were incubated with primary antibodies and protein A-agarose beads for 1 h at 4 °C. The beads were washed with RIPA buffer. Immunoprecipitated samples and 10 μg of the input sample proteins were evaluated by western blotting.

### Glutathione *S*-transferase pull-down assay

Recombinant glutathione *S*-transferase-USP21 (GST-USP21) was produced in *Escherichia coli* and used for the GST pull-down assay. The GST protein was purified using glutathione Sepharose 4B beads (Solarbio, Beijing, China) and then incubated with lysates of transfected HEK293T cells. The unbound proteins were removed by washing the beads three times with IP lysis buffer and retained proteins collected for western blotting.

### Chromatin immunoprecipitation

Chromatin immunoprecipitation (ChIP) analysis was performed using an EZ ChiP Kit (Merck Millipore, Bedford, MA, USA) according to the manufacturer’s instructions and as previously published^31^. Briefly, formaldehyde (1% final concentration; 10 min at room temperature) was used for fixation, followed by 0.125 M glycine treatment to stop the fixation reaction. The A549 cells were further centrifuged (700 × *g* for 5 min at 4 °C). The pellets were then treated with lysis buffer containing 1× protease inhibitor. The cells were sonicated to shear the DNA, and the cell debris was centrifuged at 14,000 × *g* for 10 min at 4 °C. The samples were then incubated with anti-YY1 antibody or normal rabbit immunoglobulin G (IgG) overnight at 4 °C. Immunocomplexes were then mixed with a 50% protein G agarose suspension, which was followed by incubation for 1 h. Beads were then collected by centrifugation, and the complexes were eluted with 100 mM NaHCO_3_ and 1% SDS. Chromatin was then uncrosslinked for 5 h at 65 °C. After treatment with RNase A and proteinase K, DNA was purified using spin columns and eluted with elution buffer. The primers used were as follows: forward, AGACGTGATTCCGCTTGGAG and reverse, CCCAAATCACACGGGCAAAG (product length: 443 bp).

### RNA-binding protein immunoprecipitation assay

The RNA-binding protein immunoprecipitation (RIP) experiment was performed using a Magna RIP Kit (Millipore). A total of 100 μL of whole-cell extract was incubated with RIP buffer containing magnetic beads conjugated to human anti-Argonaute2 (Ago2) antibody (Millipore) or normal mouse IgG (negative control) for 6–8 h at 4 °C. After incubation with proteinase K at 55 °C for 30 min, immunoprecipitated RNA was isolated, purified, and subjected to qRT-PCR analysis.

### Ubiquitination assay

In vivo ubiquitination were performed as previously described^32^ using Ni-NTA beads. The A549 cells were transfected with combinations of pCMV-YY1, pcDNA3-His_6_-ubiquitin, and the appropriate USP21 expression plasmid. At 44 h post transfection, 10 μM MG132 was added to each plate, and they were incubated for 4 h at 37 °C. The cells were washed twice using PBS and lysed with 1 mL of a solution containing 8 M urea, 0.1 M Na_2_HPO_4_, and 0.01 M Tris-HCl, pH 8.0. Protein quantification was performed, and protein levels of the lysate were normalized. Lysates were further incubated with Ni^2+^-NTA agarose beads and blotted for YY1.

### Electrophoretic mobility shift assay

Electrophoretic mobility shift assays (EMSAs) were conducted using the LightShift Chemiluminescence EMSA Kit (Pierce Biotechnology, Rockford, IL, USA) according to the manufacturer’s instructions and as previously described^19^. Biotin-labeled double-stranded oligonucleotides were used as competitor probes, and mutated oligonucleotides were used as negative controls. Nuclear protein was extracted from cells, and an antibody against YY1 was used to supershift the DNA–protein complex.

### Nude mouse tumor xenograft model

To investigate tumor formation in vivo, USP21- and YY1-overexpressing plasmids were packaged into a lentiviral vector, and si-USP21 and si-YY1 were independently inserted into recombinant adenoviruses. The different stably expressing H460 cells (2.5 × 10^6^) or control cells were resuspended in 200 μL of serum-free RPMI and subcutaneously injected into the flanks of male BALB/c-nu/nu mice. There were five mice in each group. Three weeks after injection, the mice were sacrificed, and tumor formation was measured. All animal care and experimental procedures were carried out in accordance with the US National Institute of Health Guidelines for Use of Experimental Animals and were approved by the Animal Ethics Committee of Shanghai Jiao Tong University (Shanghai, China).

### Statistical analysis

The results are expressed as the mean ± standard deviation. IBM SPSS statistical software for Windows, version 13.0 software (SPSS, Chicago, IL, USA) was used for statistical analysis. The comparisons between groups were performed using Student’s *t* test. A value of *p* < 0.05 was considered statistically significant.

## Results

### USP21 is overexpressed in NSCLC

**Fig. 1:**
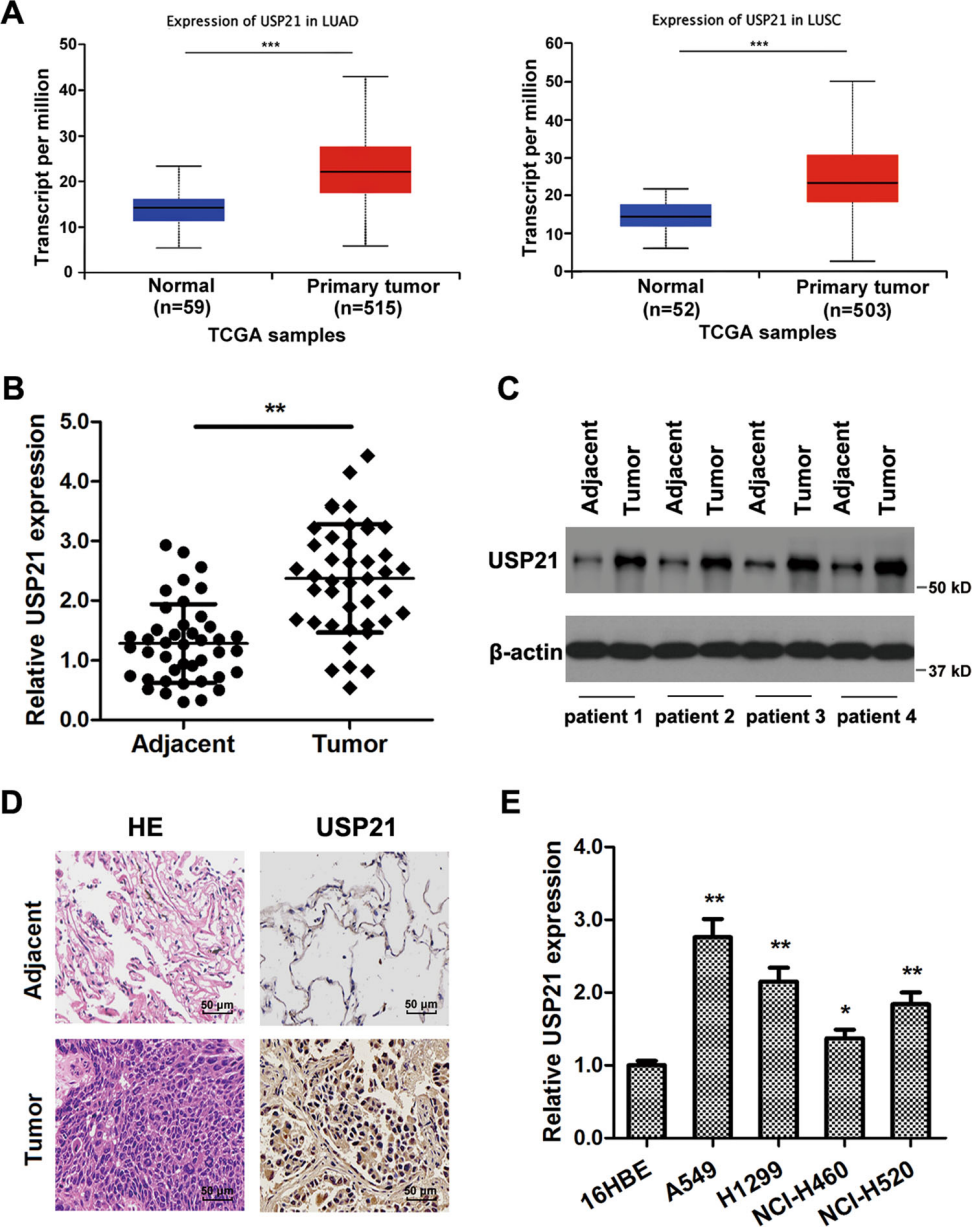
USP21 is overexpressed in non-small-cell lung cancer (NSCLC) tissues and cells. **a** Transcript levels of USP21 in lung cancer patients and normal tissues using the LUAD and LUSC data set of The Cancer Genome Atlas. **b** The relative USP21 expression levels of 42 paired lung cancer tissue samples compared to adjacent normal tissues. **c** Western blot analyses of the samples for USP21 with adjacent normal tissue samples as the controls. β-Actin was used as an internal control. **d** Immunohistochemical staining of tissue samples for USP21 with adjacent normal tissue samples as the controls. Scale bar = 50 μm. **e** The relative expression levels of USP21 in multiple NSCLC cell lines, A549, H1299, NCI-H520, and NCI-H460 and normal control 16HBE cells. The means ± SD (*n* = 3) are shown in **b**, **e**. *p < 0.05, **p < 0.01, ***p < 0.001 vs. the corresponding control.

To understand the role of DUBs in NSCLCs, we analyzed the expression levels of USP21 in LC patients using the lung adenocarcinoma (LUAD) and lung squamous cell carcinoma (LUSC) data set of The Cancer Genome Atlas (TCGA). The results showed that USP21 was overexpressed in LC tissues when compared to adjacent normal tissues (*p* < 0.01) (Fig. 1a). To validate the TCGA analyses, we measured the messenger RNA (mRNA) expression levels of USP21 in 42 paired NSCLC samples and their adjacent normal tissues, all of which were obtained from Xin Hua Hospital. Furthermore, the mRNA expression of USP21 was measured by real-time PCR. Subsequent analysis showed that USP21 was overexpressed in LC tissues when compared to the adjacent normal tissues, thus confirming our initial observation from the datasets (*p* < 0.01) (Fig. 1b). To further confirm this expression pattern, western blotting analysis was performed to detect the expression of USP21 protein in LC tissues and paired adjacent normal tissues (*p* < 0.01) (Fig. 1c). IHC analysis also showed stronger staining of USP21 in NSCLC tumor tissues than adjacent tissues (Fig. 1d). Next, we examined USP21 expression in NSCLC cell lines. The results demonstrated a higher expression of USP21 in A549, H1299, NCI-H460, and NCI-H520 cell lines in comparison to what was observed in the 16HBE normal lung cell line (Fig. 1e).

### USP21 promotes cell proliferation in vitro

**Fig. 2:**
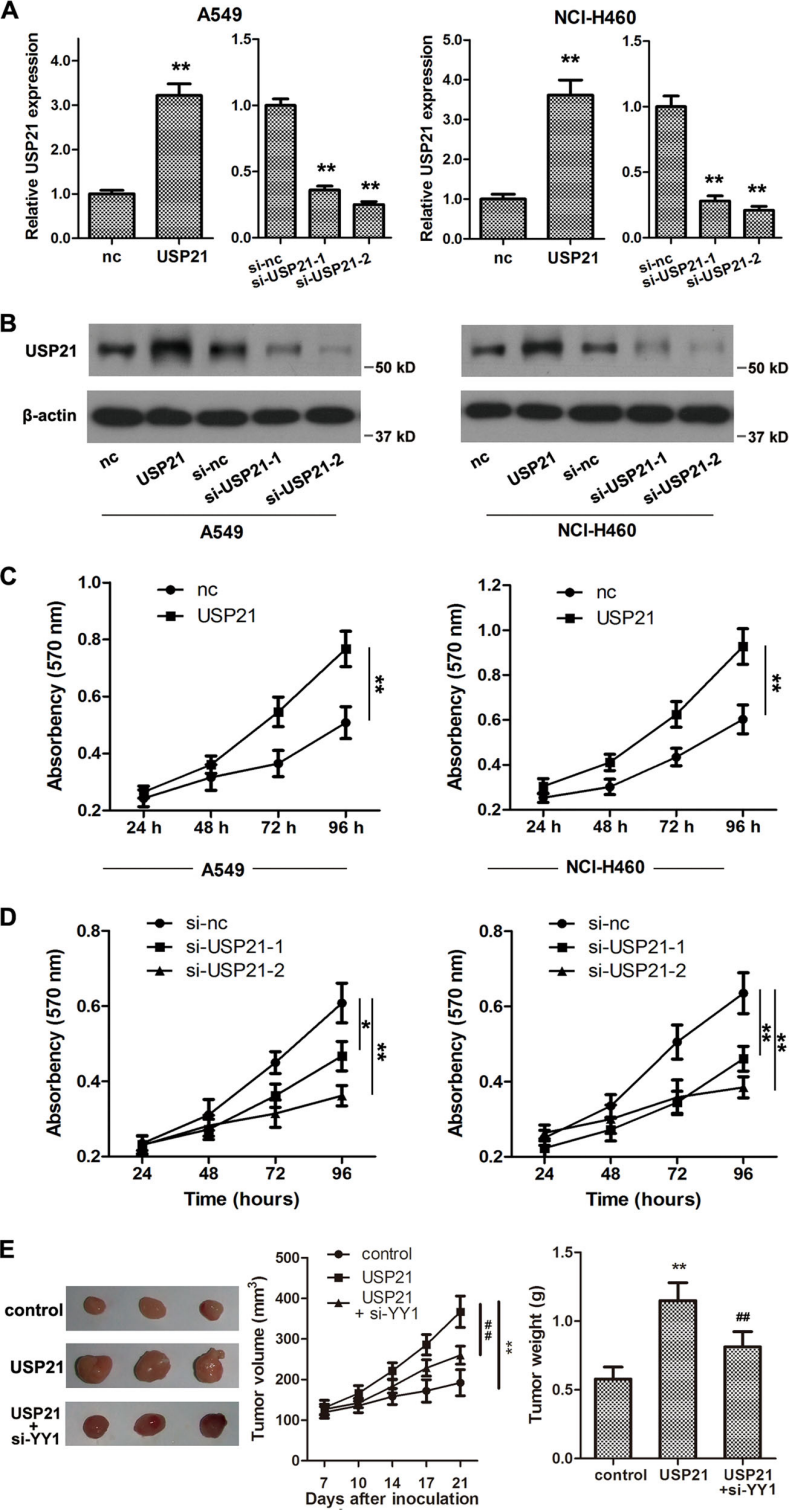
USP21 promotes cell proliferation in vitro. **a** Relative mRNA expression of USP21 after overexpression or silencing of USP21 compared with an empty vector control (nc) in A549 and NCI-H460 cells; *n* = 3. **b** Western blot analyses of USP21 after overexpression or silencing of USP21 compared with an empty vector control (nc). β-Actin was used as an internal control. **c** Cell proliferation was measured using an MTT assay at 24, 48, 72, and 96 h after transfection with the overexpression USP21 vector compared with the empty vector in A549 and NCI-H460 cells. **d** Cell proliferation was determined using the MTT assay at 24, 48, 72, and 96 h after silencing of USP21 or treating with the empty vector in A549 and NCI-H460 cells. **e** Representative images of tumor samples after 21 days of inoculation in nude mice models. Tumor weight was measured at the endpoint post 21 days, and the tumor volume was measured throughout the 21 days of inoculation. *n* = 5 mice per group in **e**. Each value is presented as the mean ± SD. *n* = 3. *p < 0.05, **p < 0.01 vs. the corresponding control; ^##^*p* < 0.01 vs. the USP21 group.

The role of USP21 in NSCLC progression was further investigated. A549 and NCI-H460 cells were selected for both USP21 silencing and overexpression experiments. We constructed a USP21 siRNA and transfected it into A549 and NCI-H460 cells. The efficiency of USP21 silencing was measured and confirmed by qPCR and western blot experiments (Fig. 2a, b). In addition, we constructed a USP21 overexpression plasmid and found that following its transfection into A549 and NCI-H460 cells, USP21 was significantly overexpressed (Fig. 2a, b). The role of USP21 in NSCLC progression was then investigated using the MTT assay. USP21 overexpression significantly promoted cell proliferation (Fig. 2c), whereas USP21 silencing inhibited cell proliferation (Fig. 2d). To investigate the effect of USP21 on tumor growth in vivo, we inoculated mice with NSCLC cells overexpressing USP21. Tumor volume and tumor weight were significantly increased in mice inoculated with USP21-overexpressing cells (Fig. 2e).

### USP21 promotes NSCLC migration and invasion

**Fig. 3:**
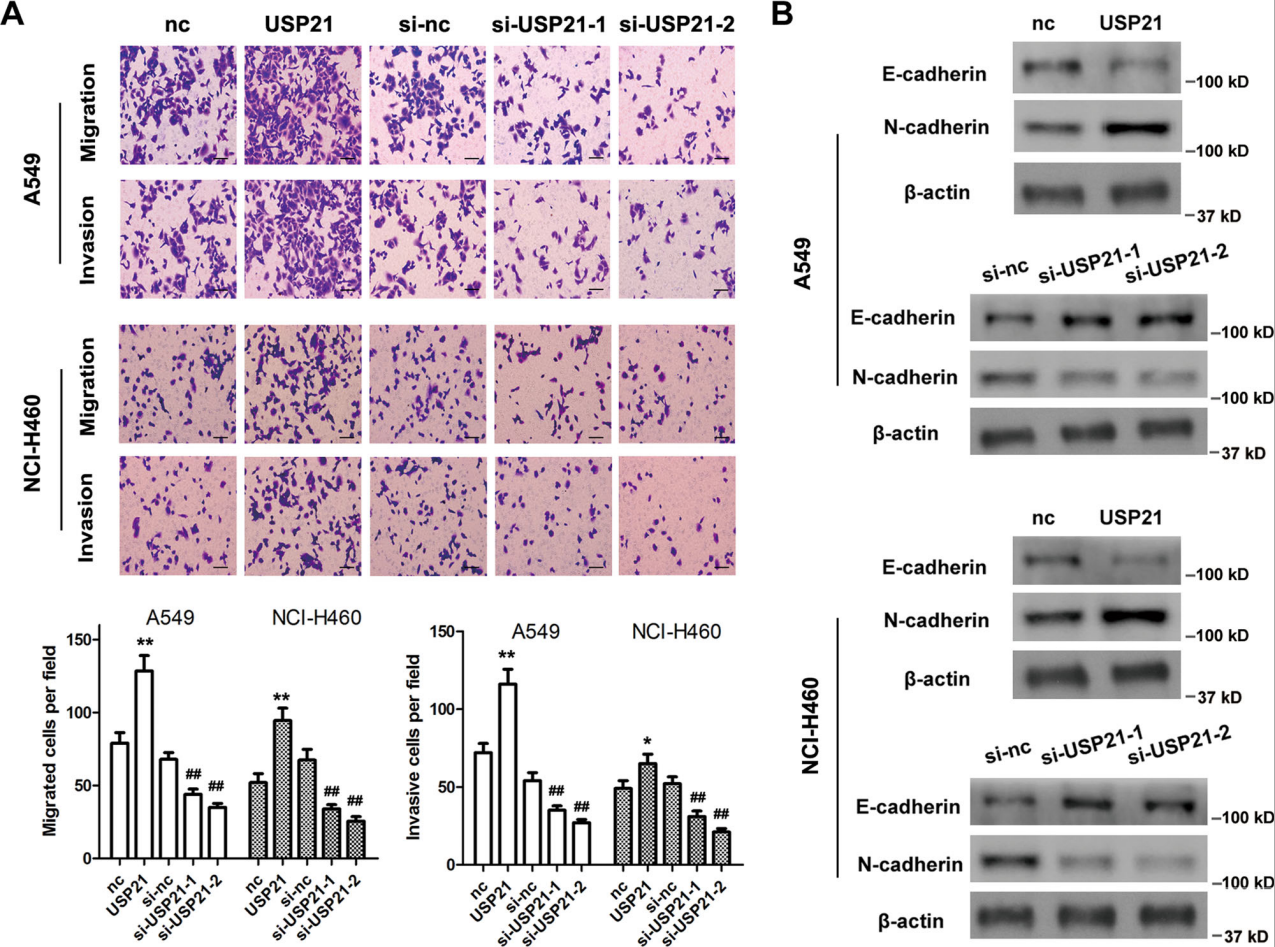
USP21 promotes NSCLC cell migration and invasion. **a** Migration and invasion of A549 and NCI-H460 cells after overexpression or silencing of USP21. Representative images (upper panel) and quantifications (lower panel) are shown. Scale bar = 100 μm. Error bars: mean ± SD. *n* = 3. *p < 0.05, **p < 0.01 vs. the normal control (nc) group. ^##^*p* < 0.01 vs. si-nc. **b** E-cadherin and N-cadherin western blot analyses after overexpression or silencing of USP21. β-Actin was used as an internal control.

The roles of USP21 in NSCLC migration and invasion were then investigated by performing transwell assays and by assessing the epithelial–mesenchymal transition (EMT)-related proteins E-cadherin and N-cadherin in A549 and NCI-H460 cells. USP21 overexpression significantly promoted cell migration and invasion, whereas USP21 silencing inhibited cell migration and invasion (Fig. 3a). Moreover, E-cadherin was significantly downregulated and N-cadherin was upregulated by USP21 overexpression. However, E-cadherin was significantly upregulated and N-cadherin was downregulated by USP21 silencing (Fig. 3b).

### USP21 plays its role as an oncogene through YY1

**Fig. 4:**
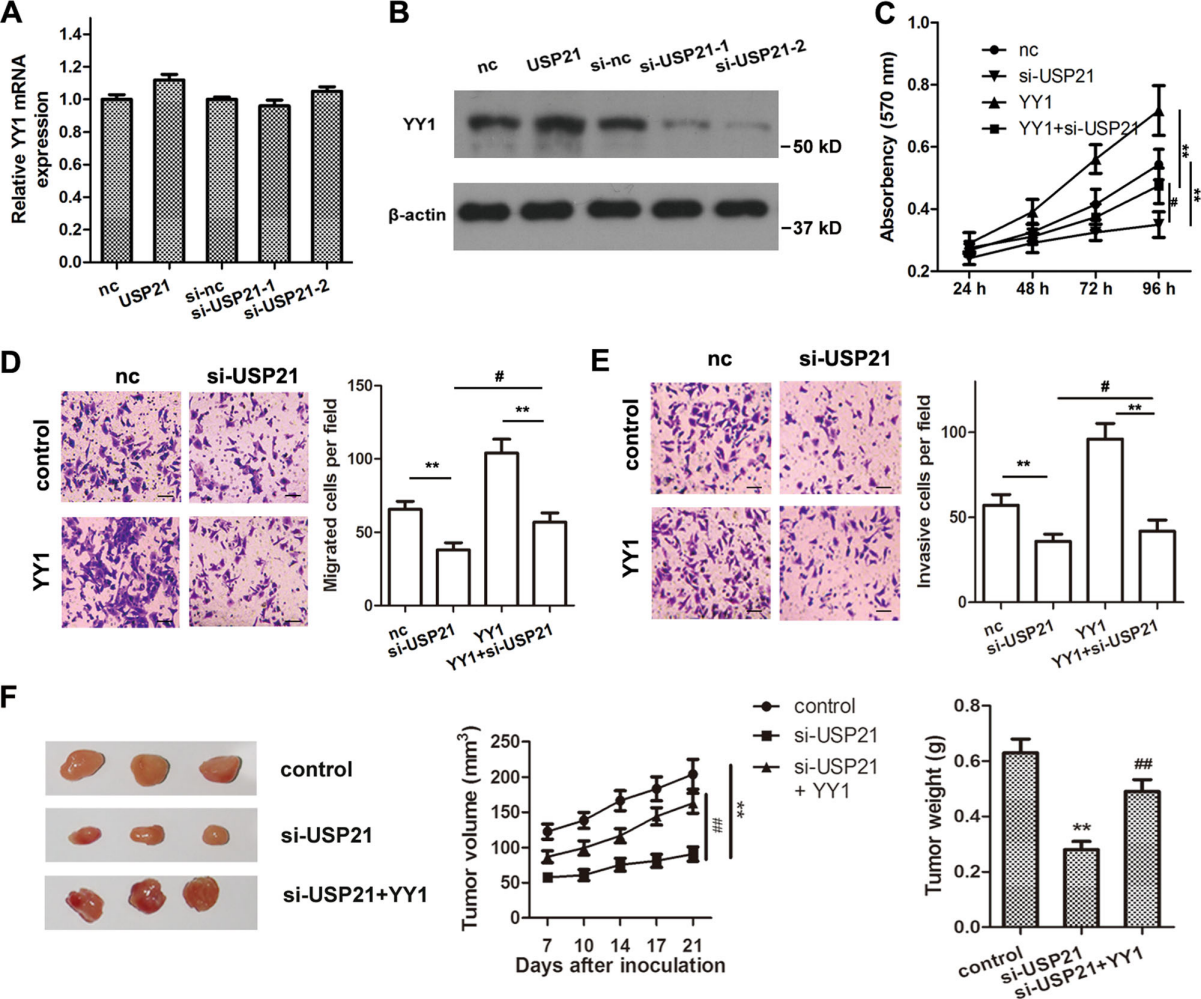
USP21 plays a role as an oncogene through Yin Yang-1 (YY1). **a** Relative YY1 mRNA expression after upregulating or silencing of USP21. **b** Western blot analyses of YY1 expression after upregulating or silencing of USP21. β-Actin was used as an internal control. **c** Cell proliferation using the MTT assay at 24, 48, 72, and 96 h in A549 cells transfected with si-USP21- and/or YY1-overexpressing plasmid. **d**, **e** Migration and invasion assays in A549 cells transfected with si-USP21- and/or YY1-overexpressing plasmid. Representative images (left panel) and quantifications (right panel) are shown. Scale bar = 100 μm. **f** The tumor weights and volumes from untransfected H460 cells (control), H460 cells transfected with si-USP21 or YY1, and H460 cells cotransfected with si-USP21 and YY1. *n* = 5 mice per group. Error bars: mean ± SD. *n* = 3. **p < 0.01 vs. the corresponding control; ^#^*p* < 0.05, ^##^*p* < 0.01 vs. the si-USP21 group.

Previous studies have shown that YY1 is involved in LC progression^19,33–35^. However, it is unknown whether the role USP21 plays as an oncogene is mediated by YY1. A USP21-overexpressing plasmid or two USP21 siRNAs were transfected into A549 cells. The results showed that USP21 overexpression did not increase the mRNA expression of YY1, but did significantly increase the levels of YY1 protein. Moreover, USP21 silencing did not decrease the expression of YY1 mRNA, but did significantly decrease the protein levels of YY1 (Fig. 4a, b). These results indicate that USP21 might be involved in the regulation of YY1 post-translational modifications. To confirm whether YY1 was involved in the oncogenic role of USP21, A549 cells were cotransfected with USP21 siRNA and a YY1-overexpressing vector. The results showed that overexpression of YY1 restored USP21 silencing inhibited cell proliferation, migration, and invasion (Fig. 4c–e). In vivo tumor xenograft model assays indicated that YY1 knockdown abolished USP21 overexpression-induced tumor growth (Fig. 2e). The rescue effects were also observed in the si-USP21 + YY1 group mice (Fig. 4f), demonstrating that USP21 may act as an oncogene through YY1.

### USP21 deubiquitinates YY1 in LC cells

**Fig. 5:**
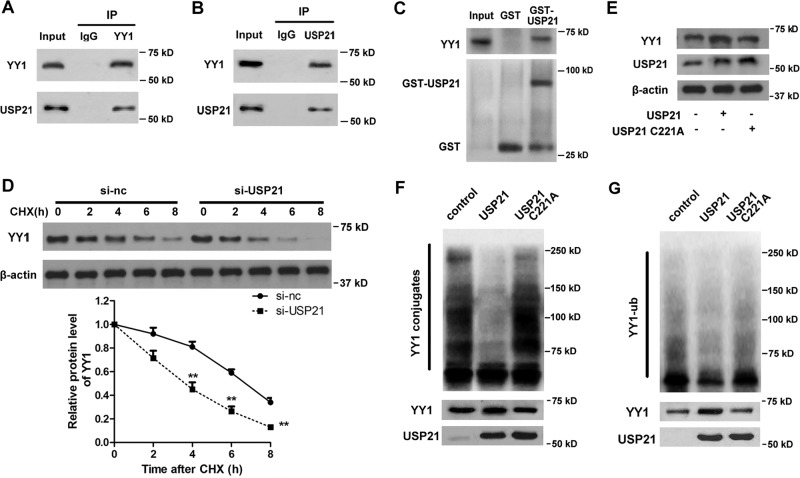
USP21 deubiquitinates YY1 in lung cancer cells. **a**, **b** The cell lysates from A549 cells were used in immunoprecipitation experiments with an anti-YY1 (**a**) or an anti-USP21 antibody (**b**) and immunoblotted with anti-YY1 and anti-USP21 antibodies, respectively. **c** GST pull-down analysis was used to determine whether USP21 directly interacted with YY1 in vitro. **d** Western blot analyses of YY1 expression in A549 cells transfected with the indicated plasmids and treated with cycloheximide. Quantification of YY1 protein levels relative to β-actin is shown. **e** Expression of YY1 in the presence of wild-type (wt) or a mutated version of USP21. β-Actin was used as an internal control. **f** Western blot analyses of YY1 conjugates. **g** Western blot analyses of ubiquitinated proteins. Statistical significance was determined by a two-tailed, unpaired Student’s *t* test. Error bars: mean ± SD. *n* = 3. **p < 0.01.

Because USP21 is a DUB, we examined whether USP21 could directly interact with and deubiquitinate YY1. USP21 was found to bind to YY1, as indicated by co-immunoprecipitation (co-IP) assays (Fig. 5a, b) and GST pull-downs (Fig. 5c), indicating that USP21 is a direct binding partner for YY1. As DUBs are involved in the ubiquitin-proteasome system, we next studied the effect of USP21 on YY1 stabilization. A549 cells were treated with cycloheximide to inhibit protein synthesis and were then transfected with USP21 siRNA. The results showed that USP21 silencing decreased the stability of YY1 and increased its degradation (Fig. 5d). Thus, we speculated that USP21 may have an effect on YY1 ubiquitination.

A previous study has shown that mutation of the His residue in the active site of the conserved His box of DUBs renders them catalytically inactive^36^. YY1 levels were then assessed in wild-type (wt) USP21 or a catalytic site mutant USP21 (USP21 C221A)-transfected cells. The results showed that cells transfected with the wt USP21 but not the USP21 C221A increased the relative levels of YY1 (Fig. 5e). To further confirm that YY1 was a deubiquitination substrate of USP21, the effect of USP21 on YY1 ubiquitination was examined. MG132 was used to inhibit degradation of ubiquitinated proteins, and the wt USP21 but not the catalytic site mutant was shown to be responsible for the reduction in YY1 conjugates (Fig. 5f). The high-molecular-weight conjugates observed were then purified and subjected to blotting for YY1, and the blots demonstrated that wt USP21 but not USP21 C221A reduced YY1 ubiquitination (Fig. 5g). Collectively, these findings suggest that USP21 deubiquitinates YY1 in LC cells.

### YY1 transcriptionally activates SNHG16 via binding to its promoter region

**Fig. 6:**
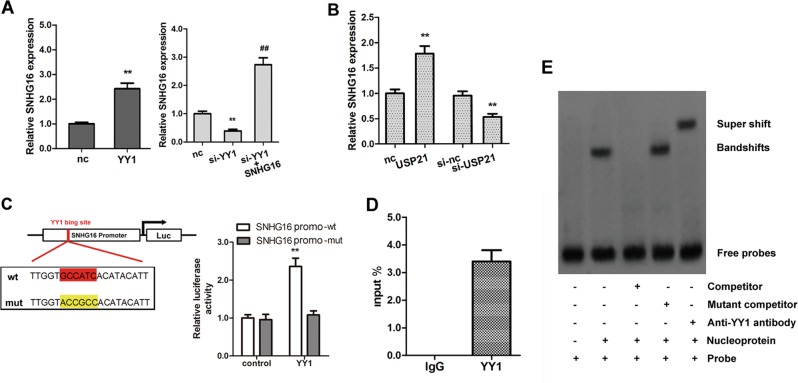
YY1 transcriptionally activates SNHG16 via binding to its promoter region. **a** Relative SNHG16 expression was measured in A549 cells with overexpressed or silenced YY1. **b** Relative expression of SNHG16 after overexpression and silencing of USP21. **c** Dual luciferase assay results using the YY1 vector and either the wt or mutant SNHG16 promoter luciferase reporter vector. **d** Chromatin immunoprecipitation assay using an antibody against YY1; isolated DNA was amplified by PCR. **e** In vitro electrophoretic mobility shift assay using the nuclear extracts and the DNA probes containing the predicted YY1 binding sites. Data are shown as the mean ± SD (*n* = 3). **p < 0.01 vs. the corresponding control; ^##^*p* < 0.01 vs. the si-YY1 group.

A previous study showed that lncRNA SNHG16 was highly expressed in NSCLC patients^29^. Previous studies have also shown that YY1 regulates lncRNA expression by binding to promoter regions and acting as a transcription factor. We were therefore interested in determining whether YY1 could affect SNHG16 expression, so we determined the levels of SNHG16 after the expression of YY1 was altered in A549 cells. We constructed a YY1-overexpressing plasmid and YY1 siRNA and then transfected them into A549 cells. The results showed that YY1 overexpression significantly increased the expression of SNHG16, whereas YY1 silencing significantly decreased the expression of SNHG16 (Fig. 6a). In addition, USP21 overexpression significantly increased SNHG16 expression, whereas silencing of USP21 significantly decreased the expression of SNHG16 (Fig. 6b) as well as YY1. With the aid of JASPAR (http://jaspar.genereg.net/cgi-bin/jaspar_db.pl), we predicted that there was a binding site for YY1 in the promoter region of lncRNA SNHG16 (Fig. 6c). The interaction between YY1 and SNHG16 was then investigated. The promoter region of SNHG16 containing the putative binding site of YY1 (wt) and a mutant (mut) form were amplified and cloned into the pmirGlo luciferase reporter vector. The wt and mutant constructs were transfected alone or with the YY1 expression vector into A549 cells. The results of dual luciferase reporter assays showed that the promoter activity of wt SNHG16 was significantly increased after transient transfection; however, no changes were observed in cells transfected with the SNHG16 mutant (Fig. 6c). To further investigate whether SNHG16 was a direct target gene of YY1, we performed ChIP assays using an antibody against YY1 and then amplified the co-purified DNA by real-time PCR. The results suggested that the SNHG16 promoter region was significantly amplified in the YY1-immunoprecipitated A549 chromatin; however, the promoter region was not observed in chromatin immunoprecipitated by the control IgG (Fig. 6d). Next, we performed EMSAs using nuclear extracts and DNA probes containing the predicted YY1 binding sites. Figure 6e shows that when DNA probes were incubated with nuclear extracts from A549 cells, a specific DNA–protein complex was observed (Fig. 6e, lane 2), and this complex was shifted to a higher position in the presence of the anti-YY1 antibody (Fig. 6e, lane 5). Taken together, these results showed that SNHG16 is a direct target gene of YY1.

### SNHG16 regulates USP21 expression by targeting miR-4500

**Fig. 7:**
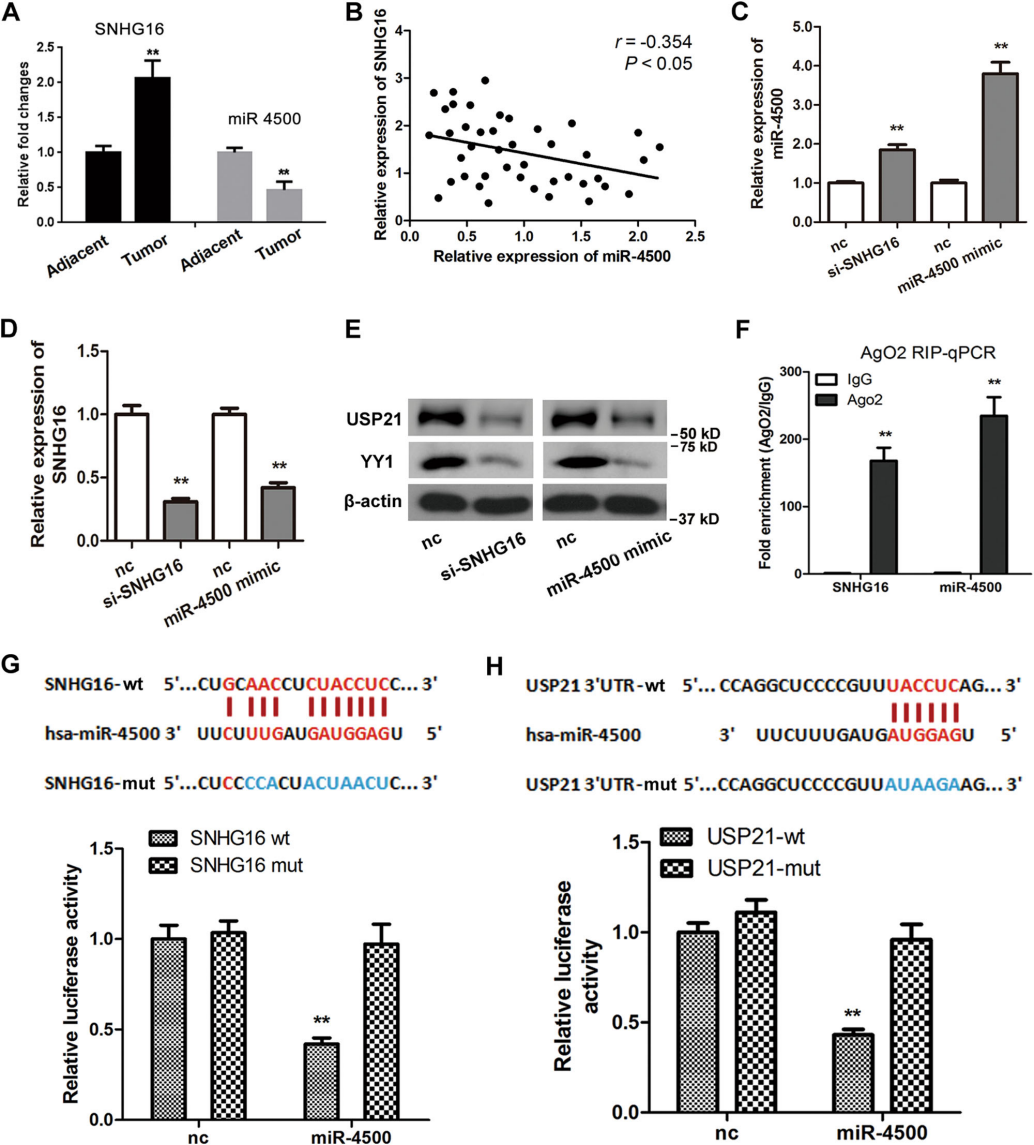
SNHG16 regulates USP21 expression by targeting miR-4500. **a** Relative expression of lncRNA SNHG16 and miR-4500 in tumor and adjacent control tissues. **b** Linear regression correlation analyses of lncRNA SNHG16 and miR-4500 expression. **c**, **d** Relative expression of miR-4500 and SNHG16 in A549 cells transfected with si-SNHG16 or miR-4500 mimic. **e** Western blot analyses of USP21 and YY1 when SNHG16 was silenced or miR-4500 mimics were used. β-Actin was used as an internal control. **f** RNA-binding protein immunoprecipitation assays with Ago2 antibodies and fold enrichment analyses of SNHG16 and miR-4500. **g**, **h** Wild-type and mutated SNHG16 (SNHG16-wt and SNHG16-mut containing the designed mutant sequence in the predicted binding sites of miR-4500) or USP21 3′-UTR (USP21 3′-UTR-wt and USP21 3′-UTR-mut containing the designed mutant sequence in the predicted binding sites of miR-4500) luciferase reporter gene vectors were constructed. The indicated vectors were cotransfected into A549 cells with miR-4500 mimics, and the luciferase activity was then determined using dual luciferase assays. Error bars: mean ± SD. *n* = 3. **p < 0.01 vs. the corresponding control.

The lncRNA SNHG16 expression levels were significantly increased in tumor tissues compared to tumor-adjacent normal tissues (*p* < 0.01) (Fig. 7a). Moreover, the miR-4500 expression levels were significantly decreased in tumor tissues compared to tumor-adjacent normal tissues (Fig. 7a). Linear regression correlation analysis results showed that the expression levels of SNHG16 were negatively associated with miR-4500 expression levels (*p* < 0.05, *r* = −0.354) (Fig. 7b). As previously mentioned, lncRNAs may act as ceRNAs or molecular sponges to modulate miRNAs, so we were interested in determining whether SNHG16 acted on miR-4500 in this way. We therefore constructed an SNHG16 siRNA and transfected it into A549 cells. The results showed that SNHG16 silencing (Fig. 7d) significantly increased the expression of miR-4500 (Fig. 7c) as well as the expression of USP21 and YY1 (Fig. 7e), while treatment with the miR-4500 mimic exerted the same effect as SNHG16 silencing (Fig. 7c–e). For further verification, we performed RIP assays with Ago2 antibodies. SNHG16 and miR-4500 were enriched in fractions associated with the Ago2-containing beads, indicating that miR-4500 was involved in SNHG16-mediated USP21 modulation (Fig. 7f). Analyses with the Starbase bioinformatics tool^37,38^ suggested that both SNHG16 and USP21 directly interacted with miR-4500 (Fig. 7g, h). To confirm our previous results, we performed luciferase reporter assays. In the presence of miR-4500, the luciferase activity was greatly decreased from reporters carrying either wt SNHG16 or wt USP21, showing that there was potentially direct binding and regulation of SNHG16 and USP21 by miR-4500 (Fig. 7g, h). Taken together, these findings suggest that lncRNA SNHG16 counteracts miR-4500 to promote USP21 expression.

## Discussion

DUBs have recently been identified for their contributions to many types of cancers^39–43^. USPs have specifically been reported to play roles in cancers such as prostate cancer (USP2a, USP7, USP10)^39–41^, ovarian cancer (USP15, USP36)^42,43^, glioblastoma (USP9X)^44^, and colorectal cancer (USP4, USP7)^45,46^. Notably, specific USPs such as USP7 regulate the turnover of many tumor suppressor proteins such as p53^47^, but USP7 also regulates Hdm2 and Hdmx, which are known to be negative regulators of p53^48^. In the current study, we identified USP21 for the first time as a DUB that is highly prevalent in NSCLC patients. We also showed that USP21 contributed to the progression of tumorigenesis by increasing proliferation, migration, and invasion. The inhibition of USP21 might therefore serve as a promising therapeutic approach in NSCLC treatment. A study by Chen et al.^10^ reported that USP21 promoted cell proliferation and metastasis in bladder cancer via deubiquitinating EZH2 and stabilizing it. EZH2 has been known to contribute to metastasis and the EMT transition, but in NSCLC, there has been no mechanism reported to date. In NSCLC, it has been reported that 25% of patients overexpressed USP1^49^, and another study has shown that inhibiting USP1 reverses resistance to cisplatin in LC cells in vitro^50^. Hence, it can be speculated that inhibition of USP21 might serve as a promising therapeutic approach in NSCLC treatment.

In our study, additional experiments showed that USP21 regulated the expression of an important oncogene, YY1. We observed that USP21 deubiquitinated YY1 and thus stabilized it. Based on previous studies, YY1, a zinc transcription factor, could act as an activator or repressor to regulate gene expression^51,52^ of many other genes. One of YY1’s identified roles is the regulation of the lncRNA LINC00152, which then promotes progression of breast cancer by affecting the stability of the PTEN protein^53^. Similarly, another study showed that YY1 binds to miR-7-5p and increased stemness in glioblastoma cells^54^. Hence, YY1 is an important player in the molecular mechanism of USP21’s role in NSCLC. Furthermore, we wanted to determine the role of noncoding RNAs regulated by YY1 in NSCLC. We found that lncRNA SNHG16 was highly upregulated when YY1 was overexpressed. The JASPAR program predicted that the promoter of lncRNA SNHG16 had a binding site for YY1, and it was shown by qPCR, ChIP, and luciferase reporter assays that YY1 transcriptionally activated SNHG16 via interaction with its promoter. The concept of genes transcriptionally activating lncRNAs and leading to tumorigenesis is well studied. It has been reported that Oct4 transcriptionally activates NEAT1 via association with its promoter and MALAT1 via enhancer binding; these actions result in increased cell proliferation and motility, which leads to lung tumorigenesis and poor prognosis^55^. In addition, another study has shown that YY1 interacts with the promoter region of lncRNA-PVT1 via its consensus YY1 motif to activate transcription in LC^19^.

LncRNAs have attracted extensive attention due to their roles in competitively binding miRNAs and regulating the expression of various mRNAs^56^. Studies have also shown that lncRNAs are associated with development and the poor prognosis of many different types of cancers^19^. LncRNA 1308 has been found to promote cell invasion by inhibiting anti-tumor miR-124 in NSCLC^57^. Other lncRNAs, such as lncRNA MIR31HG^58^, lncRNA FEZF1-AS1^59^, lncRNA-PVT1^60^, and NR2F2-AS1^61^, have been shown to play multiple roles in enhancing proliferation, the EMT transition, and metastasis in NSCLC. Furthermore, another important trait of lncRNAs is their ability to be ceRNAs, which bind and regulate miRNA. As previously mentioned, a study showed that lncRNA NR2F2-AS1 plays an important role in NSCLC^61^. More specifically, NR2F2-AS1 acts as a ceRNA by binding to miR-320b, and BMI1 is a direct target of miR-320b, which contributes to tumorigenesis. SNHG16 upregulation has been reported in NSCLC, and it also has a positive role in NSCLC carcinogenesis^29^. Zhang et al.^30^ confirmed that low expression of miR-4500 promoted tumor growth by targeting LIN28B and NRAS in NSCLC_._ In the present study, we identified miR-4500 as a novel target of SNHG16. To determine if there was direct binding between SNHG16 and miR-4500, we conducted luciferase reporter assays and RIP analyses in vitro. We verified that SNHG16 directly bound to miR-4500, which is a part of a ceRNA regulatory network^62^. In the RIP assay, both SNHG16 and miR-4500 were observed in the RNA-induced silencing complex (RISC). These combined results suggest that there was a reciprocal repression between SNHG16 and miR-4500, which was mediated by the RISC complex. However, a pivotal role for SNHG16 and miR-4500 in vivo requires further investigation. We also observed that miR-4500 bound to and inhibited USP21. Hence, SNHG16 competitively bound to miR-4500 to regulate the expression of USP21.

**Fig. 8:**
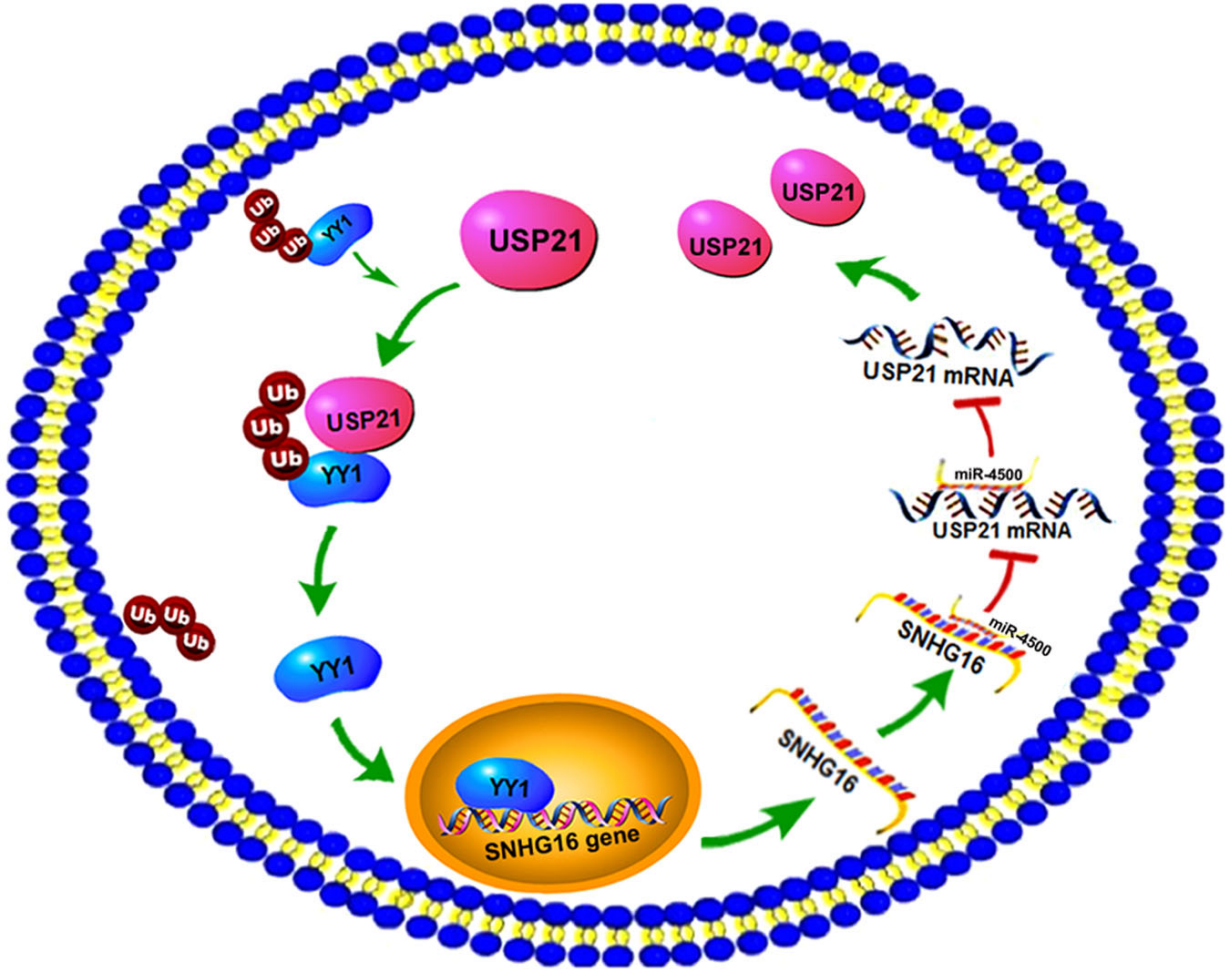
Schematic diagram of the USP21/YY1/SNHG16/miR-4500 axis. USP21 deubiquitinates and stabilizes YY1, and YY1 transcriptionally activates lncRNA SNHG16, which further upregulates USP21 expression by binding to and suppressing miR-4500.

In the current study, we showed that oncogenic USP21 deubiquitinates and stabilizes YY1 and that YY1 transcriptionally activates lncRNA SNHG16, which further acts as a ceRNA to regulate USP21 (Fig. 8). Based on the results of our study, we propose that the USP21-YY1-SNHG16 axis plays a vital role in regulating the malignant behavior of NSCLC cells. However, whether USP21/YY1/SNHG16 plays a cancer-promoting role in a feedback loop needs to be further studied. In summary, for the first time, our study has identified the contribution of the USP21/YY1/SNHG16/miR-4500 axis in NSCLC, which might provide therapeutic strategies for the treatment of NSCLC.

## References

[1] Chen W (2016). Cancer statistics in China, 2015. CA Cancer J. Clin..

[2] Chen W (2017). Cancer incidence and mortality in China in 2013: an analysis based on urbanization level. Chin. J. Cancer Res..

[3] Sibille A (2015). Management of non-small cell lung cancer. Rev. Med. Liege.

[4] Ma PC (2012). Personalized targeted therapy in advanced non-small cell lung cancer. Cleve. Clin. J. Med..

[5] Torre LA, Siegel RL, Jemal A (2016). Lung cancer statistics. Adv. Exp. Med. Biol..

[6] Ye Y (2011). Polyubiquitin binding and cross-reactivity in the USP domain deubiquitinase USP21. EMBO Rep..

[7] Pannu J (2015). Ubiquitin specific protease 21 is dispensable for normal development, hematopoiesis and lymphocyte differentiation. PLoS ONE.

[8] Nakagawa T (2008). Deubiquitylation of histone H2A activates transcriptional initiation via trans-histone cross-talk with H3K4 di- and trimethylation. Genes Dev..

[9] Peng L, Hu Y, Chen D, Jiao S, Sun S (2016). Ubiquitin specific peptidase 21 regulates interleukin-8 expression, stem-cell like property of human renal cell carcinoma. Oncotarget.

[10] Chen Y, Zhou B, Chen D (2017). USP21 promotes cell proliferation and metastasis through suppressing EZH2 ubiquitination in bladder carcinoma. OncoTargets Ther..

[11] Li W, Cui K, Prochownik EV, Li Y (2018). The deubiquitinase USP21 stabilizes MEK2 to promote tumor growth. Cell Death Dis..

[12] Zhang J (2013). Identification of the E3 deubiquitinase ubiquitin-specific peptidase 21 (USP21) as a positive regulator of the transcription factor GATA3. J. Biol. Chem..

[13] Heride C (2016). The centrosomal deubiquitylase USP21 regulates Gli1 transcriptional activity and stability. J. Cell Sci..

[14] Shi Y, Seto E, Chang LS, Shenk T (1991). Transcriptional repression by YY1, a human GLI-Kruppel-related protein, and relief of repression by adenovirus E1A protein. Cell.

[15] Gordon S, Akopyan G, Garban H, Bonavida B (2006). Transcription factor YY1: structure, function, and therapeutic implications in cancer biology. Oncogene.

[16] Khachigian LM (2018). The Yin and Yang of YY1 in tumor growth and suppression. Int. J. Cancer.

[17] Kang W (2014). Yin Yang 1 contributes to gastric carcinogenesis and its nuclear expression correlates with shorter survival in patients with early stage gastric adenocarcinoma. J. Transl. Med..

[18] Kashyap V, Bonavida B (2014). Role of YY1 in the pathogenesis of prostate cancer and correlation with bioinformatic data sets of gene expression. Genes Cancer.

[19] Huang T (2017). Transcription factor YY1 modulates lung cancer progression by activating lncRNA-PVT1. DNA Cell Biol..

[20] Lin C, Yang L (2018). Long noncoding RNA in cancer: wiring signaling circuitry. Trends Cell Biol..

[21] Salmena L, Poliseno L, Tay Y, Kats L, Pandolfi PP (2011). A ceRNA hypothesis: the Rosetta Stone of a hidden RNA language?. Cell.

[22] Zhang Z, Fu C, Xu Q, Wei X (2017). Long non-coding RNA CASC7 inhibits the proliferation and migration of colon cancer cells via inhibiting microRNA-21. Biomed. Pharmacother..

[23] Liu F (2016). Long noncoding RNA FTX inhibits hepatocellular carcinoma proliferation and metastasis by binding MCM2 and miR-374a. Oncogene.

[24] Cao X, Xu J, Yue D (2018). LncRNA-SNHG16 predicts poor prognosis and promotes tumor proliferation through epigenetically silencing p21 in bladder cancer. Cancer Gene Ther..

[25] Cai C, Huo Q, Wang X, Chen B, Yang Q (2017). SNHG16 contributes to breast cancer cell migration by competitively binding miR-98 with E2F5. Biochem. Biophys. Res. Commun..

[26] Zhu H, Zeng Y, Zhou CC, Ye W (2018). SNHG16/miR-216-5p/ZEB1 signal pathway contributes to the tumorigenesis of cervical cancer cells. Arch. Biochem. Biophys..

[27] Lian D, Amin B, Du D, Yan W (2017). Enhanced expression of the long non-coding RNA SNHG16 contributes to gastric cancer progression and metastasis. Cancer Biomark..

[28] Lu YF (2018). LncRNA SNHG16 functions as an oncogene by sponging miR-4518 and up-regulating PRMT5 expression in Glioma. Cell Physiol. Biochem..

[29] Han W (2019). Increased expression of long non-coding RNA SNHG16 correlates with tumor progression and poor prognosis in non-small cell lung cancer. Int. J. Biol. Macromol..

[30] Zhang L (2014). Down-regulation of miR-4500 promoted non-small cell lung cancer growth. Cell Physiol. Biochem..

[31] Zhang JJ (2017). Yin Yang-1 suppresses pancreatic ductal adenocarcinoma cell proliferation and tumor growth by regulating SOX2OT-SOX2 axis. Cancer Lett..

[32] Stevenson LF (2007). The deubiquitinating enzyme USP2a regulates the p53 pathway by targeting Mdm2. EMBO J..

[33] Gao D (2018). Spleen tyrosine kinase SYK(L) interacts with YY1 and coordinately suppresses SNAI2 transcription in lung cancer cells. FEBS J..

[34] Huang T (2017). MiR-186 inhibits proliferation, migration, and invasion of non-small cell lung cancer cells by downregulating Yin Yang 1. Cancer Biomark..

[35] Zhang Y (2018). miR29a suppresses IL13induced cell invasion by inhibiting YY1 in the AKT pathway in lung adenocarcinoma A549 cells. Oncol. Rep..

[36] Yoo KJ (2005). Expression and functional analyses of mHAUSP regulating apoptosis of cervical adenocarcinoma cells. Int. J. Oncol..

[37] Yang JH (2011). starBase: a database for exploring microRNA-mRNA interaction maps from Argonaute CLIP-Seq and Degradome-Seq data. Nucleic Acids Res..

[38] Li JH, Liu S, Zhou H, Qu LH, Yang JH (2014). starBase v2.0: decoding miRNA-ceRNA, miRNA-ncRNA and protein–RNA interaction networks from large-scale CLIP-Seq data. Nucleic Acids Res..

[39] Graner E (2004). The isopeptidase USP2a regulates the stability of fatty acid synthase in prostate cancer. Cancer Cell.

[40] Song MS (2008). The deubiquitinylation and localization of PTEN are regulated by a HAUSP-PML network. Nature.

[41] Draker R, Sarcinella E, Cheung P (2011). USP10 deubiquitylates the histone variant H2A.Z and both are required for androgen receptor-mediated gene activation. Nucleic Acids Res..

[42] Padmanabhan A (2018). USP15-dependent lysosomal pathway controls p53-R175H turnover in ovarian cancer cells. Nat. Commun..

[43] Li J (2008). Differential display identifies overexpression of the USP36 gene, encoding a deubiquitinating enzyme, in ovarian cancer. Int. J. Med. Sci..

[44] Wolfsperger F (2016). Deubiquitylating enzyme USP9x regulates radiosensitivity in glioblastoma cells by Mcl-1-dependent and -independent mechanisms. Cell Death Dis..

[45] Yun SI (2015). Ubiquitin specific protease 4 positively regulates the WNT/beta-catenin signaling in colorectal cancer. Mol. Oncol..

[46] Novellasdemunt L (2017). USP7 is a tumor-specific WNT activator for APC-mutated colorectal cancer by mediating beta-catenin deubiquitination. Cell Rep..

[47] Li M (2002). Deubiquitination of p53 by HAUSP is an important pathway for p53 stabilization. Nature.

[48] Meulmeester E (2005). Loss of HAUSP-mediated deubiquitination contributes to DNA damage-induced destabilization of Hdmx and Hdm2. Mol. Cell.

[49] Garcia-Santisteban I, Peters GJ, Giovannetti E, Rodríguez JA (2013). USP1 deubiquitinase: cellular functions, regulatory mechanisms and emerging potential as target in cancer therapy. Mol. Cancer.

[50] Sourisseau T (2016). Translational regulation of the mRNA encoding the ubiquitin peptidase USP1 involved in the DNA damage response as a determinant of cisplatin resistance. Cell Cycle.

[51] Camacho-Moctezuma B, Quevedo-Castillo M, Melendez-Zajgla J, Aquino-Jarquin G, Martinez-Ruiz GU (2019). YY1 negatively regulates the XAF1 gene expression in prostate cancer. Biochem. Biophys. Res. Commun..

[52] Balkhi MY, Wittmann G, Xiong F, Junghans RP (2018). YY1 upregulates checkpoint receptors and downregulates type I cytokines in exhausted, chronically stimulated human T Cells. iScience.

[53] Shen X, Zhong J, Yu P, Zhao Q, Huang T (2019). YY1-regulated LINC00152 promotes triple negative breast cancer progression by affecting on stability of PTEN protein. Biochem. Biophys. Res. Commun..

[54] Jia B (2019). MiR-7-5p suppresses stemness and enhances temozolomide sensitivity of drug-resistant glioblastoma cells by targeting Yin Yang 1. Exp. Cell Res..

[55] Jen J (2017). Oct4 transcriptionally regulates the expression of long non-coding RNAs NEAT1 and MALAT1 to promote lung cancer progression. Mol. Cancer.

[56] Tay Y, Rinn J, Pandolfi PP (2014). The multilayered complexity of ceRNA crosstalk and competition. Nature.

[57] Li H (2018). Long non-coding RNA 1308 promotes cell invasion by regulating the miR-124/ADAM 15 axis in non-small-cell lung cancer cells. Cancer Manag. Res.

[58] Dandan W, Jianliang C, Haiyan H, Hang M, Xuedong L (2019). Long noncoding RNA MIR31HG is activated by SP1 and promotes cell migration and invasion by sponging miR-214 in NSCLC. Gene.

[59] He R, Zhang FH, Shen N (2017). LncRNA FEZF1-AS1 enhances epithelial-mesenchymal transition (EMT) through suppressing E-cadherin and regulating WNT pathway in non-small cell lung cancer (NSCLC). Biomed. Pharmacother..

[60] Guo D, Wang Y, Ren K, Han X (2018). Knockdown of LncRNA PVT1 inhibits tumorigenesis in non-small-cell lung cancer by regulating miR-497 expression. Exp. Cell Res..

[61] Zhang S (2019). LncRNA NR2F2-AS1 promotes tumourigenesis through modulating BMI1 expression by targeting miR-320b in non-small cell lung cancer. J. Cell. Mol. Med..

[62] Renganathan A, Felley-Bosco E (2017). Long noncoding RNAs in cancer and therapeutic potential. Adv. Exp. Med. Biol..

